# Supplementary material for: Development and validation of an LC-MS/MS method for determination of B vitamins and some its derivatives in whole blood
Source: PLoS One. 2022 Jul 14;17(7):e0271444. doi: 10.1371/journal.pone.0271444 (PMC9282541; doi:10.1371/journal.pone.0271444)
Supplement: S1 File — (PDF) [file pone.0271444.s001.pdf]

Entered data

Description – Calibration Solution	Val. property – Concentration [µg/L]	Measurement – Peak Area Ratio [1]
Calibration_Standard_001_µg/L	1	0.00352420967286692
Calibration_Standard_001_µg/L	1	0.00336177822353571
Calibration_Standard_001_µg/L	1	0.00342433642745021
Calibration_Standard_002.5_µg/L	2.5	0.00880357271008481
Calibration_Standard_002.5_µg/L	2.5	0.00870137771961254
Calibration_Standard_002.5_µg/L	2.5	0.00860355929554257
Calibration_Standard_005_µg/L	5	0.0191278159017026
Calibration_Standard_005_µg/L	5	0.01873682533173
Calibration_Standard_005_µg/L	5	0.0176757605507607
Calibration_Standard_010_µg/L	10	0.0378832011982676
Calibration_Standard_010_µg/L	10	0.0364688317673684
Calibration_Standard_010_µg/L	10	0.037304451784498
Calibration_Standard_025_µg/L	25	0.0967074690612784
Calibration_Standard_025_µg/L	25	0.0924441571147502
Calibration_Standard_025_µg/L	25	0.0939025246901804
Calibration_Standard_050_µg/L	50	0.187167803108529
Calibration_Standard_050_µg/L	50	0.1821693740576
Calibration_Standard_050_µg/L	50	0.192537701468484
Calibration_Standard_100_µg/L	100	0.378149942699267
Calibration_Standard_100_µg/L	100	0.381546863083519
Calibration_Standard_100_µg/L	100	0.375539306320656

Results

Calculated R	R^2	Reference R	Calculated QC	Reference QC	Max. acceptable intercept (absolute)	Calculated intercept value	Hypothesis
0.9999	0.9997	0.99	2.0453	5	20	-0.0006	Accepted

Chart

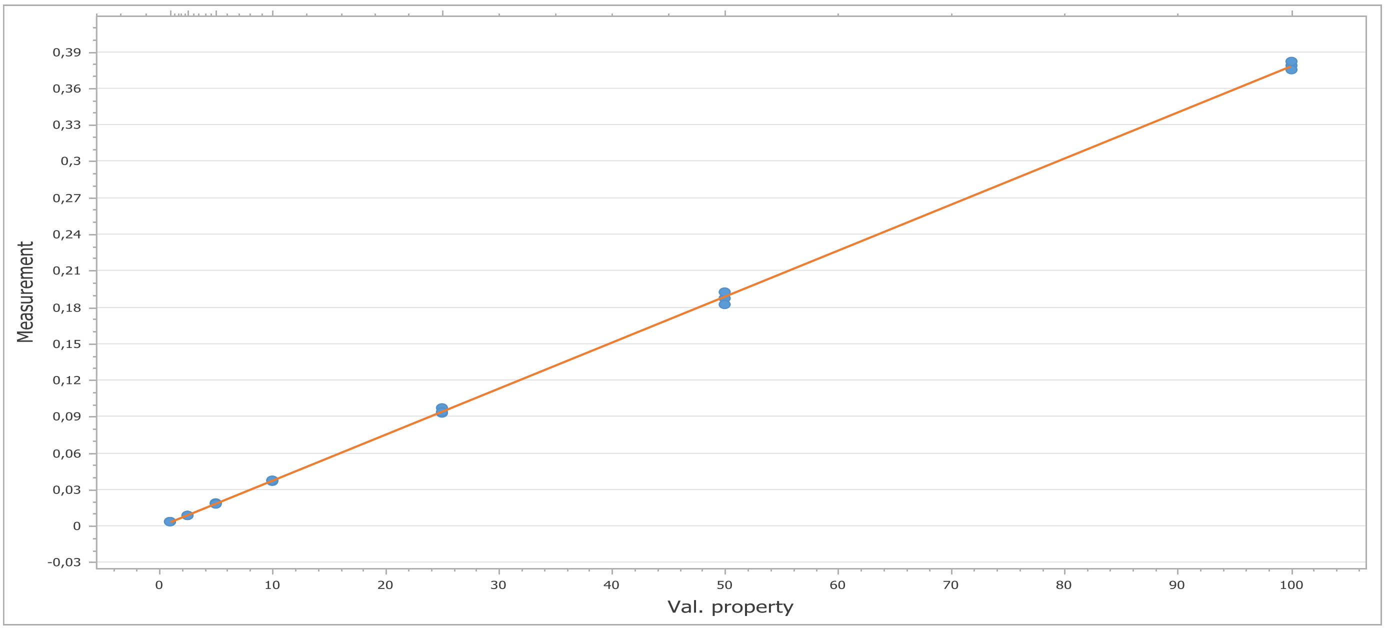

Conclusion: Linearity of the method was confirmed based on correlation and QC Coefficient.

The intercept value of the given straight line meets the acceptance criterion.

Straight line equation: y=-0.0006+0.0038*x.

Supporting statistics: Intercept (estimate)= -0.000637

Slope (estimate)= 0.003785

Intercept standard deviation (estimate)= 0.000632

Slope standard deviation (estimate)= 1.4E-05

Confidence interval for intercept= (-0.001959) - (0.000686)

Confidence interval for slope= (0.003755) - (0.003815)

Residual variance (estimate)= 5E-06

Residual standard deviation (estimate)= 0.002182

Correlation coefficient= 0.999867

R^2= 0.999734

QC coefficient= 2.045285

Entered data

Description – Calibration Solution	Val. property – Concentration [µg/L]	Measurement – Peak Area Ratio [1]
Calibration_Standard_001_µg/L	1	0.00067949680503474
Calibration_Standard_001_µg/L	1	0.000826658241392064
Calibration_Standard_001_µg/L	1	0.000817866125961785
Calibration_Standard_002.5_µg/L	2.5	0.0019673760794822
Calibration_Standard_002.5_µg/L	2.5	0.00186346822923031
Calibration_Standard_002.5_µg/L	2.5	0.00190518445329015
Calibration_Standard_005_µg/L	5	0.00363878840664161
Calibration_Standard_005_µg/L	5	0.0037553971674263
Calibration_Standard_005_µg/L	5	0.00367286773591532
Calibration_Standard_010_µg/L	10	0.0076896060031041
Calibration_Standard_010_µg/L	10	0.00742803868282474
Calibration_Standard_010_µg/L	10	0.00733869869418541
Calibration_Standard_025_µg/L	25	0.0178748265137735
Calibration_Standard_025_µg/L	25	0.0187412538107889
Calibration_Standard_025_µg/L	25	0.0178900235298996
Calibration_Standard_050_µg/L	50	0.0374735481342587
Calibration_Standard_050_µg/L	50	0.0355553552062185
Calibration_Standard_050_µg/L	50	0.0360007974296111
Calibration_Standard_100_µg/L	100	0.0737711948423214
Calibration_Standard_100_µg/L	100	0.0725628009961918
Calibration_Standard_100_µg/L	100	0.0744866673619677
Calibration_Standard_250_µg/L	250	0.1831614113633
Calibration_Standard_250_µg/L	250	0.185666043681101
Calibration_Standard_250_µg/L	250	0.182048066400845
Calibration_Standard_500_µg/L	500	0.367003584668907
Calibration_Standard_500_µg/L	500	0.36982283288788
Calibration_Standard_500_µg/L	500	0.364535537435662

Results

Calculated R	R^2	Reference R	Calculated QC	Reference QC	Max. acceptable intercept (absolute)	Calculated intercept value	Hypothesis
1	0.9999	0.99	1.3008	5	20	0	Accepted

Chart

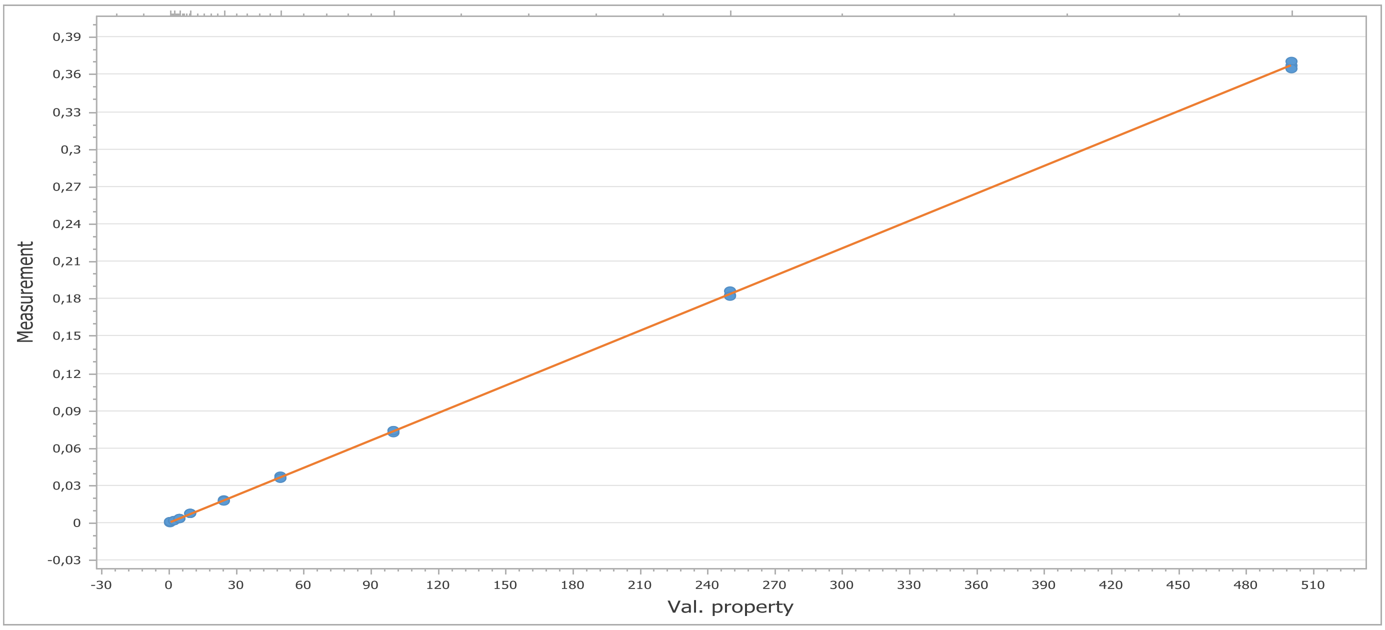

Conclusion: Linearity of the method was confirmed based on correlation and QC Coefficient.

The intercept value of the given straight line meets the acceptance criterion.

Straight line equation: y=0+0.0007*x.

Supporting statistics: Intercept (estimate)= -1.1E-05

Slope (estimate)= 0.000734

Intercept standard deviation (estimate)= 0.00024

Slope standard deviation (estimate)= 1E-06

Confidence interval for intercept= (-0.000505) - (0.000483)

Confidence interval for slope= (0.000732) - (0.000737)

Residual variance (estimate)= 1E-06

Residual standard deviation (estimate)= 0.001021

Correlation coefficient= 0.999964

R^2= 0.999929

QC coefficient= 1.300821

Entered data

Description – Calibration Solution	Val. property – Concentration [μg/L]	Measurement – Peak Area Ratio [1]
Calibration_Standard_010_μg/L	10	0.0534805424217436
Calibration_Standard_010_μg/L	10	0.0521085261292123
Calibration_Standard_010_μg/L	10	0.0568188312694424
Calibration_Standard_025_μg/L	25	0.138237361176221
Calibration_Standard_025_μg/L	25	0.132524858454847
Calibration_Standard_025_μg/L	25	0.132515231697803
Calibration_Standard_050_μg/L	50	0.281255952560169
Calibration_Standard_050_μg/L	50	0.277341597389255
Calibration_Standard_050_μg/L	50	0.274551058994486
Calibration_Standard_100_μg/L	100	0.56152339751258
Calibration_Standard_100_μg/L	100	0.553009796961609
Calibration_Standard_100_μg/L	100	0.554858525006312
Calibration_Standard_250_μg/L	250	1.44035786839489
Calibration_Standard_250_μg/L	250	1.41718581780438
Calibration_Standard_250_μg/L	250	1.40744835621654
Calibration_Standard_500_μg/L	500	2.8708188882323
Calibration_Standard_500_μg/L	500	2.85008342010687
Calibration_Standard_500_μg/L	500	2.79542862137968

Results

Calculated R	R^2	Reference R	Calculated QC	Reference QC	Max. acceptable intercept (absolute)	Calculated intercept value	Hypothesis
0.9999	0.9998	0.99	1.7303	5	20	-0.0067	Accepted

Chart

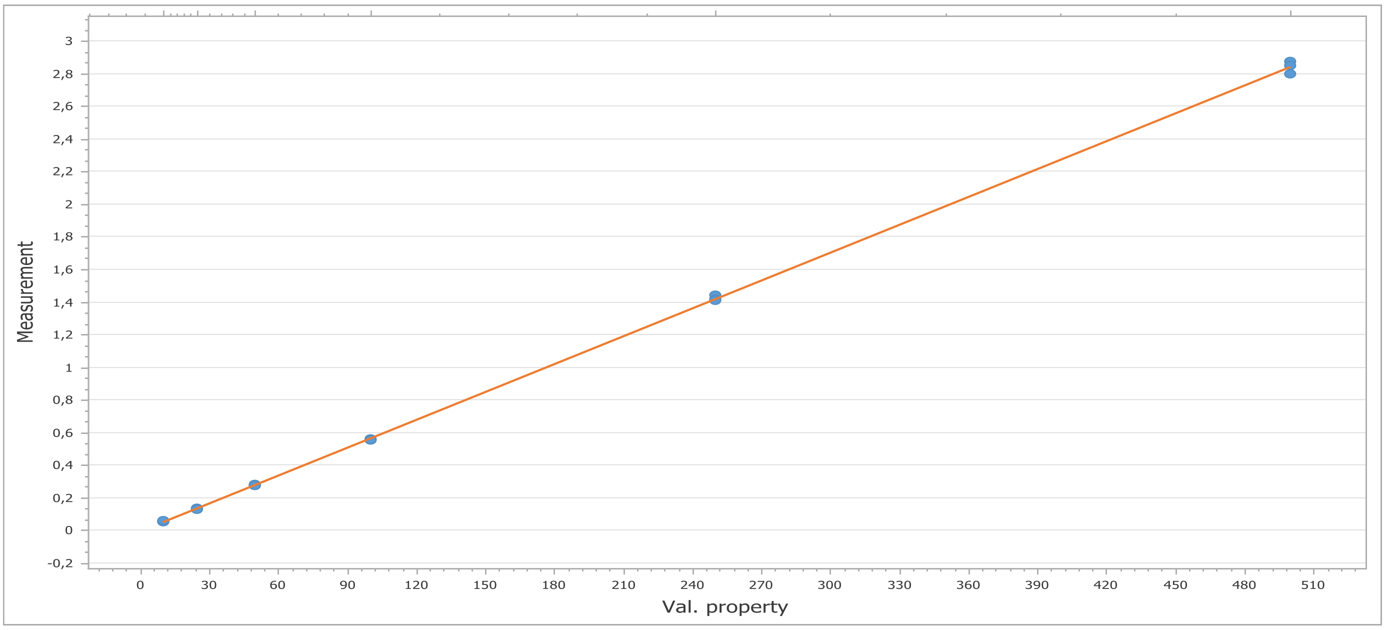

Conclusion: Linearity of the method was confirmed based on correlation and QC Coefficient.

The intercept value of the given straight line meets the acceptance criterion.

Straight line equation: y=-0.0067+0.0057*x.

Supporting statistics: Intercept (estimate)= -0.006701

Slope (estimate)= 0.005693

Intercept standard deviation (estimate)= 0.005123

Slope standard deviation (estimate)= 2.1E-05

Confidence interval for intercept= (-0.017563) - (0.00416)

Confidence interval for slope= (0.005648) - (0.005739)

Residual variance (estimate)= 0.000247

Residual standard deviation (estimate)= 0.015705

Correlation coefficient= 0.999887

R^2= 0.999775

QC coefficient= 1.730279

EffiChem Confidence in Quality	Linearity - Correlation and QC coefficient<br>B3-AM (Niacinamide)	EffiChem s.r.o.<br>Purkyňova 649/127<br>Brno

Entered data

Description – Calibration Solution	Val. property – Concentration [µg/L]	Measurement – Peak Area Ratio [1]
Calibration_Standard_005_µg/L	5	0.135576313062412
Calibration_Standard_005_µg/L	5	0.128717627025138
Calibration_Standard_005_µg/L	5	0.131840450005568
Calibration_Standard_010_µg/L	10	0.23064386358096
Calibration_Standard_010_µg/L	10	0.23855456868425
Calibration_Standard_010_µg/L	10	0.241517272615157
Calibration_Standard_025_µg/L	25	0.541922799607134
Calibration_Standard_025_µg/L	25	0.544656865911759
Calibration_Standard_025_µg/L	25	0.549607759292333
Calibration_Standard_050_µg/L	50	1.04497809486392
Calibration_Standard_050_µg/L	50	1.07726548696496
Calibration_Standard_050_µg/L	50	0.998843031861012
Calibration_Standard_100_µg/L	100	2.0087330185913
Calibration_Standard_100_µg/L	100	1.96512593607393
Calibration_Standard_100_µg/L	100	1.9345895100395
Calibration_Standard_250_µg/L	250	4.9922101173442
Calibration_Standard_250_µg/L	250	4.71821526906379
Calibration_Standard_250_µg/L	250	4.71143587998721
Calibration_Standard_500_µg/L	500	9.64062237762431
Calibration_Standard_500_µg/L	500	9.84851186495509
Calibration_Standard_500_µg/L	500	9.65934259614093

Results

Calculated R	R^2	Reference R	Calculated QC	Reference QC	Max. acceptable intercept (absolute)	Calculated intercept value	Hypothesis
0.9998	0.9995	0.99	2.6948	5	20	0.0453	Accepted

Chart

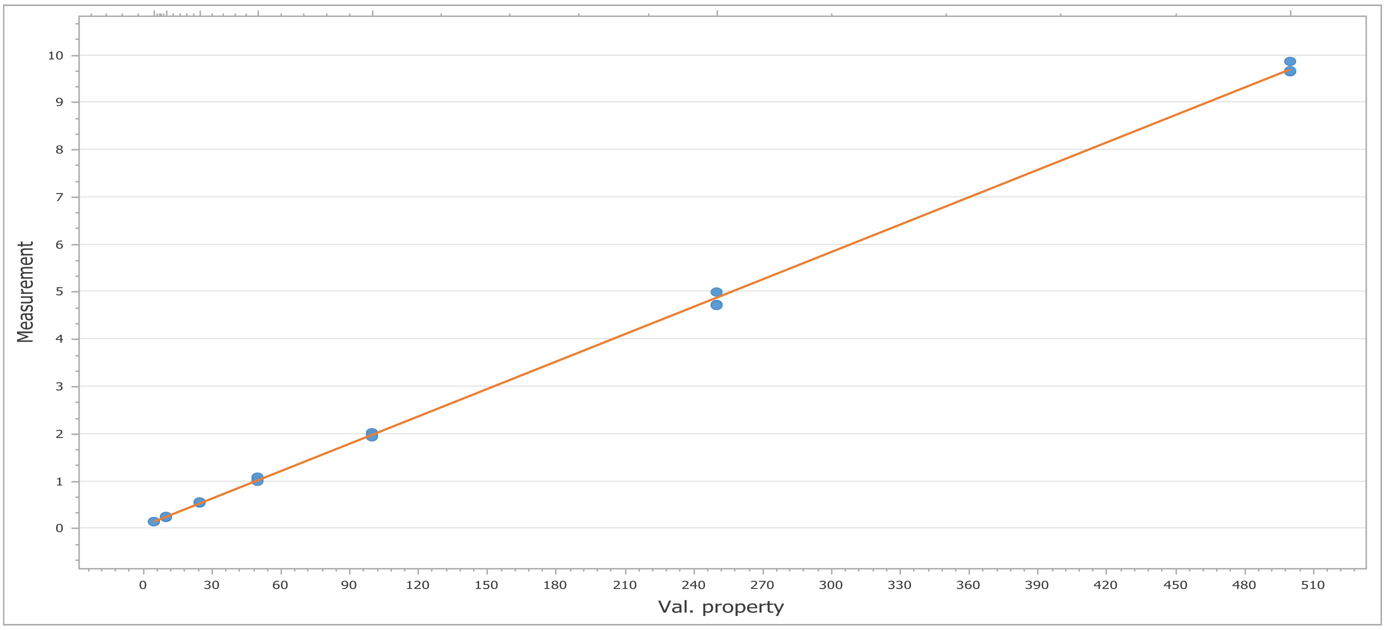

Conclusion: Linearity of the method was confirmed based on correlation and QC Coefficient.

The intercept value of the given straight line meets the acceptance criterion.

Straight line equation: y=0.0453+0.0193*x.

Supporting statistics: Intercept (estimate)= 0.045335

Slope (estimate)= 0.019288

Intercept standard deviation (estimate)= 0.020818

Slope standard deviation (estimate)= 9.4E-05

Confidence interval for intercept= (0.001763) - (0.088908)

Confidence interval for slope= (0.01909) - (0.019485)

Residual variance (estimate)= 0.005309

Residual standard deviation (estimate)= 0.072863

Correlation coefficient= 0.999774

R^2= 0.999547

QC coefficient= 2.694793

Entered data

Description – Calibration Solution	Val. property – Concentration [µg/L]	Measurement – Peak Area Ratio [1]
Calibration_Standard_002.5_µg/L	2.5	0.0111048556167835
Calibration_Standard_002.5_µg/L	2.5	0.01134267879741
Calibration_Standard_002.5_µg/L	2.5	0.010827284901178
Calibration_Standard_005_µg/L	5	0.0190335159213793
Calibration_Standard_005_µg/L	5	0.0193530497387934
Calibration_Standard_005_µg/L	5	0.0189922380223836
Calibration_Standard_010_µg/L	10	0.0385784487155503
Calibration_Standard_010_µg/L	10	0.0377334713958818
Calibration_Standard_010_µg/L	10	0.0393215102255052
Calibration_Standard_025_µg/L	25	0.0943232098499363
Calibration_Standard_025_µg/L	25	0.0945798650545513
Calibration_Standard_025_µg/L	25	0.0942782748696772
Calibration_Standard_050_µg/L	50	0.182755007847179
Calibration_Standard_050_µg/L	50	0.183612683804687
Calibration_Standard_050_µg/L	50	0.182738281459682
Calibration_Standard_100_µg/L	100	0.37392089727981
Calibration_Standard_100_µg/L	100	0.364854792155511
Calibration_Standard_100_µg/L	100	0.372189961946975
Calibration_Standard_250_µg/L	250	0.920222492598849
Calibration_Standard_250_µg/L	250	0.914659647673858
Calibration_Standard_250_µg/L	250	0.916998095093734
Calibration_Standard_500_µg/L	500	1.83560491705407
Calibration_Standard_500_µg/L	500	1.84123964916688
Calibration_Standard_500_µg/L	500	1.83754087663561

Results

Calculated R	R^2	Reference R	Calculated QC	Reference QC	Max. acceptable intercept (absolute)	Calculated intercept value	Hypothesis
1	1	0.99	0.5318	5	20	0.0014	Accepted

Chart

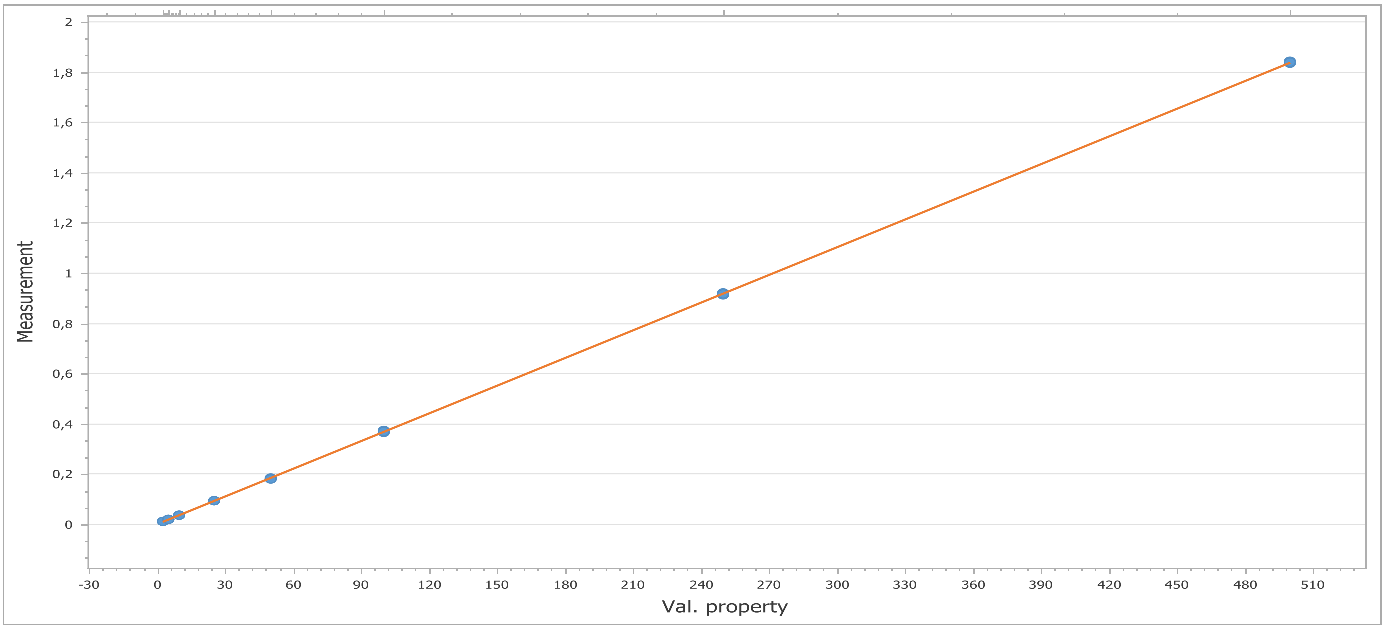

Conclusion: Linearity of the method was confirmed based on correlation and QC Coefficient.

The intercept value of the given straight line meets the acceptance criterion.

Straight line equation: y=0.0014+0.0037*x.

Supporting statistics: Intercept (estimate)= 0.001407

Slope (estimate)= 0.003672

Intercept standard deviation (estimate)= 0.000606

Slope standard deviation (estimate)= 3E-06

Confidence interval for intercept= (0.00015) - (0.002664)

Confidence interval for slope= (0.003666) - (0.003678)

Residual variance (estimate)= 6E-06

Residual standard deviation (estimate)= 0.00236

Correlation coefficient= 0.999993

R^2= 0.999986

QC coefficient= 0.531792

Entered data

Description – Calibration Solution	Val. property – Concentration [µg/L]	Measurement – Peak Area Ratio [1]
Calibration_Standard_001_µg/L	1	0.0354954969337963
Calibration_Standard_001_µg/L	1	0.0342293886653154
Calibration_Standard_001_µg/L	1	0.0341418152221134
Calibration_Standard_002.5_µg/L	2.5	0.0969043208169548
Calibration_Standard_002.5_µg/L	2.5	0.102330330734759
Calibration_Standard_002.5_µg/L	2.5	0.100672891689235
Calibration_Standard_005_µg/L	5	0.179118832973959
Calibration_Standard_005_µg/L	5	0.207122620623237
Calibration_Standard_005_µg/L	5	0.203178155480205
Calibration_Standard_010_µg/L	10	0.366910924520411
Calibration_Standard_010_µg/L	10	0.381340737202349
Calibration_Standard_010_µg/L	10	0.368213408062055
Calibration_Standard_025_µg/L	25	1.05381761835888
Calibration_Standard_025_µg/L	25	0.983553949414578
Calibration_Standard_025_µg/L	25	0.971257012811183
Calibration_Standard_050_µg/L	50	2.05455856891706
Calibration_Standard_050_µg/L	50	2.08333430290154
Calibration_Standard_050_µg/L	50	1.93706545393316
Calibration_Standard_100_µg/L	100	3.66067256161517
Calibration_Standard_100_µg/L	100	3.72251110751888
Calibration_Standard_100_µg/L	100	3.96241666637872
Calibration_Standard_250_µg/L	250	9.93179845113206
Calibration_Standard_250_µg/L	250	9.74588241776307
Calibration_Standard_250_µg/L	250	9.87919837536303
Calibration_Standard_500_µg/L	500	20.028421295836
Calibration_Standard_500_µg/L	500	19.5490930982085
Calibration_Standard_500_µg/L	500	19.4401341207283

Results

Calculated R	R^2	Reference R	Calculated QC	Reference QC	Max. acceptable intercept (absolute)	Calculated intercept value	Hypothesis
0.9998	0.9997	0.99	2.8692	5	20	-0.0097	Accepted

Chart

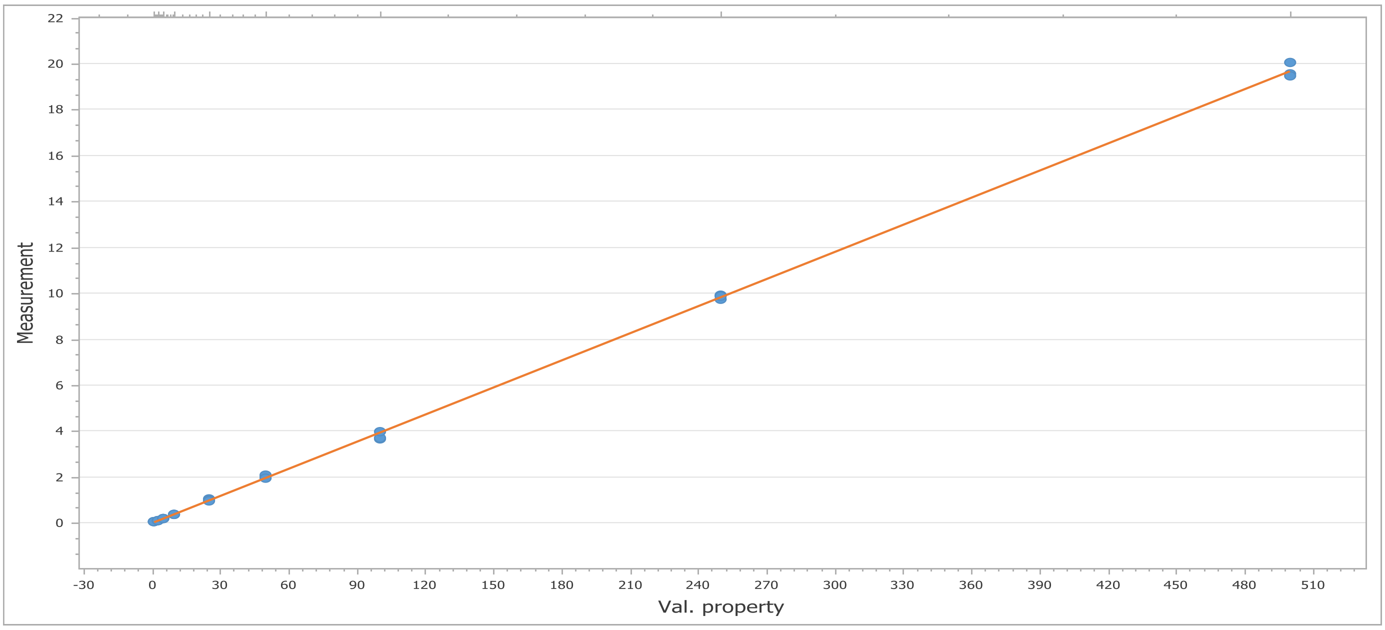

Conclusion: Linearity of the method was confirmed based on correlation and QC Coefficient.

The intercept value of the given straight line meets the acceptance criterion.

Straight line equation: y=-0.0097+0.0393*x.

Supporting statistics: Intercept (estimate)= -0.009706

Slope (estimate)= 0.039348

Intercept standard deviation (estimate)= 0.028299

Slope standard deviation (estimate)= 0.000146

Confidence interval for intercept= (-0.068003) - (0.048591)

Confidence interval for slope= (0.039048) - (0.039649)

Residual variance (estimate)= 0.0145

Residual standard deviation (estimate)= 0.120416

Correlation coefficient= 0.999828

R^2= 0.999656

QC coefficient= 2.869227

Entered data

Description – Calibration Solution	Val. property – Concentration [µg/L]	Measurement – Peak Area Ratio [1]
Calibration_Standard_005_µg/L	5	0.00314260715068839
Calibration_Standard_005_µg/L	5	0.00310317079282196
Calibration_Standard_005_µg/L	5	0.00298028248575365
Calibration_Standard_010_µg/L	10	0.00671231260908685
Calibration_Standard_010_µg/L	10	0.00659421609639534
Calibration_Standard_010_µg/L	10	0.00643321315944531
Calibration_Standard_025_µg/L	25	0.0177065445842402
Calibration_Standard_025_µg/L	25	0.0180677440861674
Calibration_Standard_025_µg/L	25	0.0167865794835273
Calibration_Standard_050_µg/L	50	0.0367231349011293
Calibration_Standard_050_µg/L	50	0.0374790106565063
Calibration_Standard_050_µg/L	50	0.0363451881252027
Calibration_Standard_100_µg/L	100	0.0799855731148707
Calibration_Standard_100_µg/L	100	0.0767620201996312
Calibration_Standard_100_µg/L	100	0.0765771095624044
Calibration_Standard_250_µg/L	250	0.202290148070223
Calibration_Standard_250_µg/L	250	0.197652952848667
Calibration_Standard_250_µg/L	250	0.200603746903299
Calibration_Standard_500_µg/L	500	0.392601615526087
Calibration_Standard_500_µg/L	500	0.39482782798708
Calibration_Standard_500_µg/L	500	0.396808599054439

Results

Calculated R	R^2	Reference R	Calculated QC	Reference QC	Max. acceptable intercept (absolute)	Calculated intercept value	Hypothesis
0.9999	0.9998	0.99	1.7609	5	20	-0.0015	Accepted

Chart

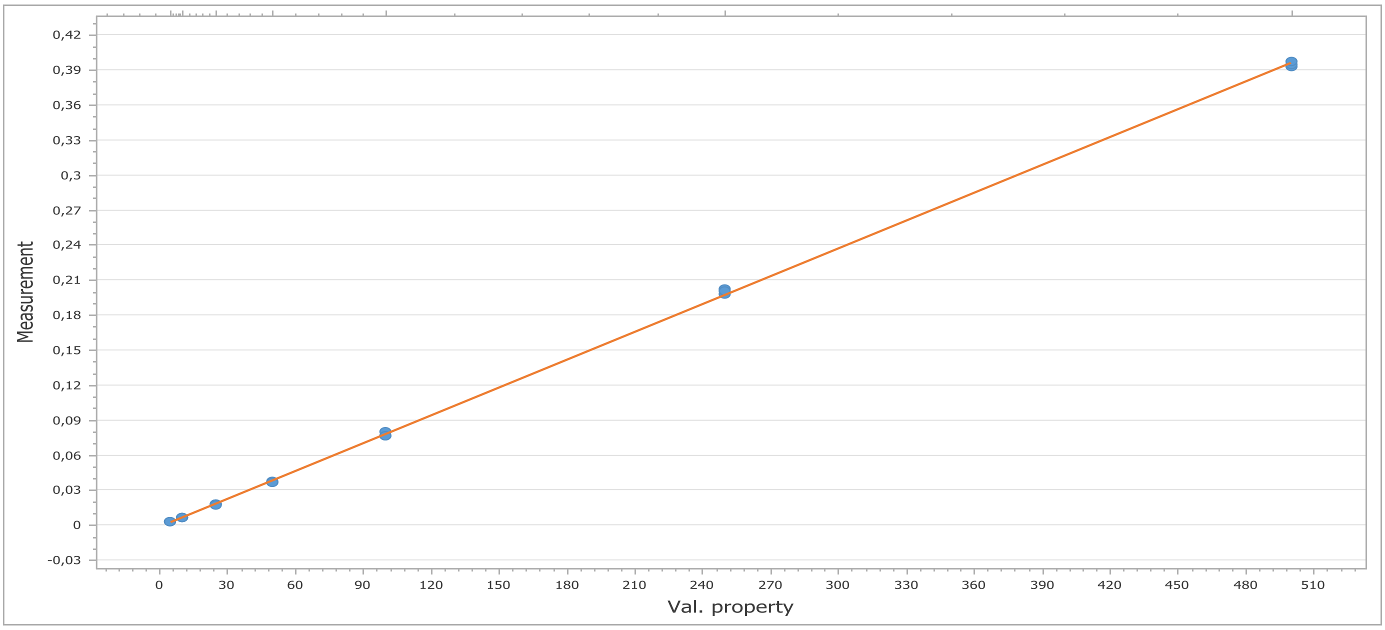

Conclusion: Linearity of the method was confirmed based on correlation and QC Coefficient.

The intercept value of the given straight line meets the acceptance criterion.

Straight line equation: y=-0.0015+0.0008*x.

Supporting statistics: Intercept (estimate)= -0.001514

Slope (estimate)= 0.000795

Intercept standard deviation (estimate)= 0.000543

Slope standard deviation (estimate)= 2E-06

Confidence interval for intercept= (-0.002651) - (-0.000377)

Confidence interval for slope= (0.00079) - (0.0008)

Residual variance (estimate)= 4E-06

Residual standard deviation (estimate)= 0.001901

Correlation coefficient= 0.999909

R^2= 0.999818

QC coefficient= 1.76086

	Linearity - Correlation and QC coefficient<br>B6-4PA (4-Pyridoxic acid)	EffiChem s.r.o.<br>Purkyňova 649/127<br>Brno

Entered data

Description – Calibration Solution	Val. property – Concentration [µg/L]	Measurement – Peak Area Ratio [1]
Calibration_Standard_001_µg/L	1	0.0238066126443071
Calibration_Standard_001_µg/L	1	0.0206407366425974
Calibration_Standard_001_µg/L	1	0.0200639883197801
Calibration_Standard_002.5_µg/L	2.5	0.050280167851156
Calibration_Standard_002.5_µg/L	2.5	0.0515143216187237
Calibration_Standard_002.5_µg/L	2.5	0.0500809024078272
Calibration_Standard_005_µg/L	5	0.0856863731204358
Calibration_Standard_005_µg/L	5	0.090719054701818
Calibration_Standard_005_µg/L	5	0.0924296797322188
Calibration_Standard_010_µg/L	10	0.16297399331002
Calibration_Standard_010_µg/L	10	0.174183643141146
Calibration_Standard_010_µg/L	10	0.16749918209186
Calibration_Standard_025_µg/L	25	0.387313037583545
Calibration_Standard_025_µg/L	25	0.409464149319182
Calibration_Standard_025_µg/L	25	0.398953535409165
Calibration_Standard_050_µg/L	50	0.859904633699983
Calibration_Standard_050_µg/L	50	0.825036025800485
Calibration_Standard_050_µg/L	50	0.814775544751228
Calibration_Standard_100_µg/L	100	1.66895700608554
Calibration_Standard_100_µg/L	100	1.6122259011589
Calibration_Standard_100_µg/L	100	1.6465944089692

Results

Calculated R	R^2	Reference R	Calculated QC	Reference QC	Max. acceptable intercept (absolute)	Calculated intercept value	Hypothesis
0.9997	0.9993	0.99	3.1659	5	20	0.0048	Accepted

Chart

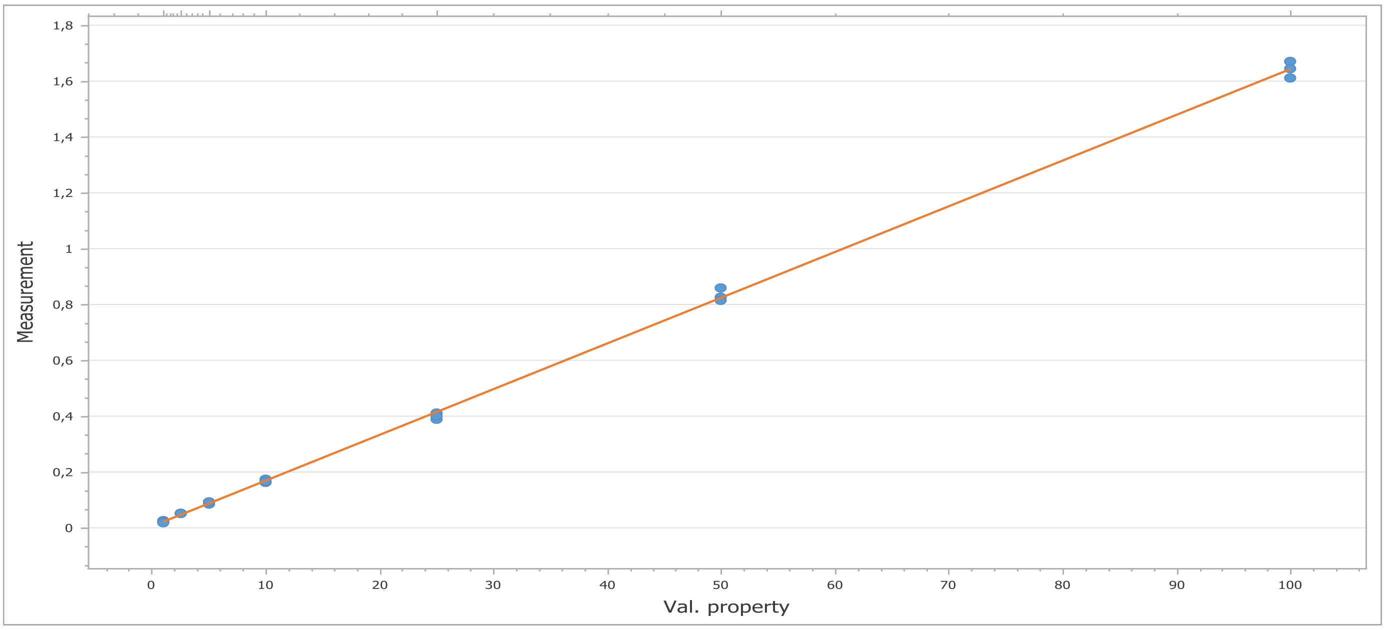

Conclusion: Linearity of the method was confirmed based on correlation and QC Coefficient.

The intercept value of the given straight line meets the acceptance criterion.

Straight line equation: y=0.0048+0.0164*x.

Supporting statistics: Intercept (estimate)= 0.004812

Slope (estimate)= 0.016386

Intercept standard deviation (estimate)= 0.004305

Slope standard deviation (estimate)= 9.7E-05

Confidence interval for intercept= (-0.004198) - (0.013822)

Confidence interval for slope= (0.016184) - (0.016588)

Residual variance (estimate)= 0.000221

Residual standard deviation (estimate)= 0.014869

Correlation coefficient= 0.99967

R^2= 0.999341

QC coefficient= 3.16588

Entered data

Description – Calibration Solution	Val. property – Concentration [µg/L]	Measurement – Peak Area Ratio [1]
Calibration_Standard_001_µg/L	1	0.00382242381053133
Calibration_Standard_001_µg/L	1	0.00367432931473915
Calibration_Standard_001_µg/L	1	0.00368578756571579
Calibration_Standard_002.5_µg/L	2.5	0.00901883286801451
Calibration_Standard_002.5_µg/L	2.5	0.00894305006839559
Calibration_Standard_002.5_µg/L	2.5	0.00898244661175238
Calibration_Standard_005_µg/L	5	0.0177107218033038
Calibration_Standard_005_µg/L	5	0.0173609515819469
Calibration_Standard_005_µg/L	5	0.0170617825993446
Calibration_Standard_010_µg/L	10	0.0318556683900235
Calibration_Standard_010_µg/L	10	0.0323742088953014
Calibration_Standard_010_µg/L	10	0.0331018743493365
Calibration_Standard_025_µg/L	25	0.0813219122314418
Calibration_Standard_025_µg/L	25	0.0811156984198693
Calibration_Standard_025_µg/L	25	0.0751894018741628
Calibration_Standard_050_µg/L	50	0.155801722086477
Calibration_Standard_050_µg/L	50	0.159783439432182
Calibration_Standard_050_µg/L	50	0.155454018538239
Calibration_Standard_100_µg/L	100	0.305034390891427
Calibration_Standard_100_µg/L	100	0.306270253911347
Calibration_Standard_100_µg/L	100	0.310130873091
Calibration_Standard_250_µg/L	250	0.783708176290854
Calibration_Standard_250_µg/L	250	0.771989169740879
Calibration_Standard_250_µg/L	250	0.783667300121485
Calibration_Standard_500_µg/L	500	1.544987002955
Calibration_Standard_500_µg/L	500	1.56483965701252
Calibration_Standard_500_µg/L	500	1.54877156785035

Results

Calculated R	R^2	Reference R	Calculated QC	Reference QC	Max. acceptable intercept (absolute)	Calculated intercept value	Hypothesis
1	0.9999	0.99	1.2711	5	20	0.001	Accepted

Chart

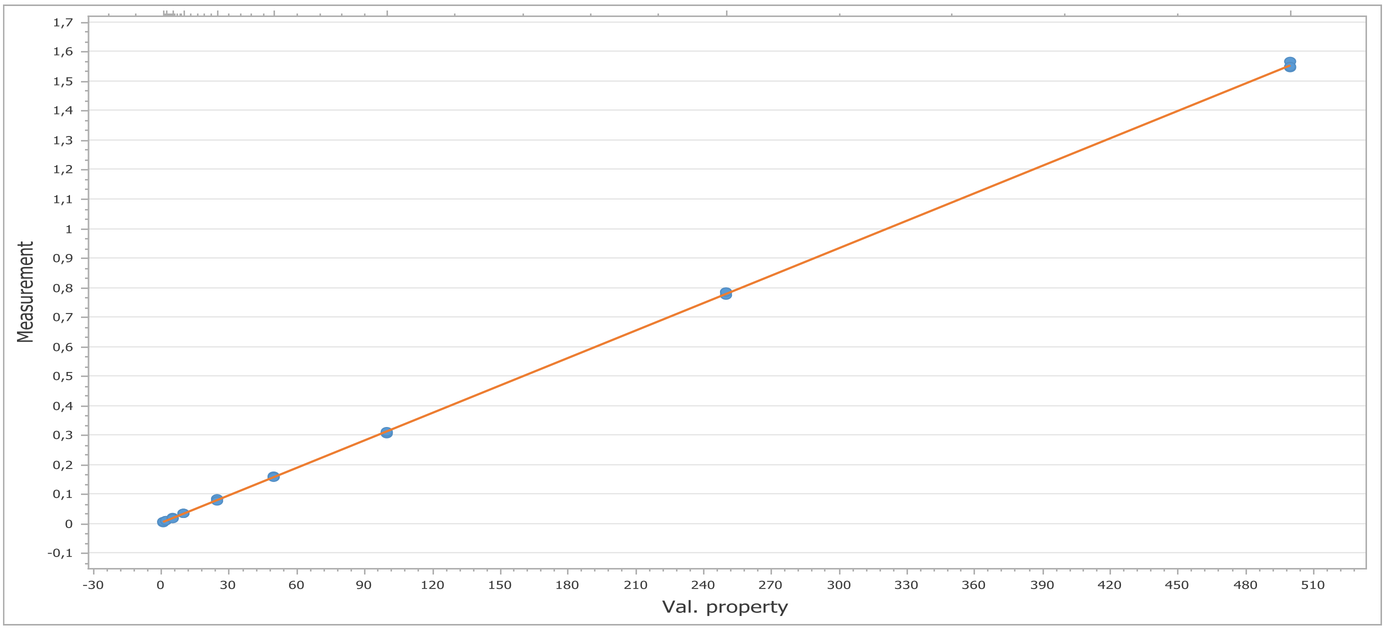

Conclusion: Linearity of the method was confirmed based on correlation and QC Coefficient.

The intercept value of the given straight line meets the acceptance criterion.

Straight line equation: y=0.001+0.0031*x.

Supporting statistics: Intercept (estimate)= 0.001024

Slope (estimate)= 0.003105

Intercept standard deviation (estimate)= 0.000995

Slope standard deviation (estimate)= 5E-06

Confidence interval for intercept= (-0.001025) - (0.003073)

Confidence interval for slope= (0.003094) - (0.003115)

Residual variance (estimate)= 1.8E-05

Residual standard deviation (estimate)= 0.004232

Correlation coefficient= 0.999966

R^2= 0.999932

QC coefficient= 1.271058

Entered data

Description – Calibration Solution	Val. property – Concentration [µg/L]	Measurement – Peak Area Ratio [1]
Calibration_Standard_001_µg/L	1	5,10301016042416E-05
Calibration_Standard_001_µg/L	1	5,55618394060633E-05
Calibration_Standard_001_µg/L	1	5,02906423319449E-05
Calibration_Standard_002.5_µg/L	2,5	0,000101776113149417
Calibration_Standard_002.5_µg/L	2,5	0,000114647177892368
Calibration_Standard_002.5_µg/L	2,5	0,000108700073029118
Calibration_Standard_005_µg/L	5	0,000184579760815194
Calibration_Standard_005_µg/L	5	0,000197993676743434
Calibration_Standard_005_µg/L	5	0,000201233687648175
Calibration_Standard_010_µg/L	10	0,000343090987727154
Calibration_Standard_010_µg/L	10	0,000363996461243611
Calibration_Standard_010_µg/L	10	0,000372025774180333
Calibration_Standard_025_µg/L	25	0,000900592605046846
Calibration_Standard_025_µg/L	25	0,000843294106483874
Calibration_Standard_025_µg/L	25	0,000806440249655097
Calibration_Standard_050_µg/L	50	0,00162389761075021
Calibration_Standard_050_µg/L	50	0,00173025668885529
Calibration_Standard_050_µg/L	50	0,00170073401280075
Calibration_Standard_100_µg/L	100	0,00341787472271494
Calibration_Standard_100_µg/L	100	0,00334642142096616
Calibration_Standard_100_µg/L	100	0,0033018676166793

Results

Calculated R	R^2	Reference R	Calculated QC	Reference QC	Max. acceptable intercept (absolute)	Calculated intercept value	Hypothesis
0,9997	0,9993	0,99	3,2139	5	20	0	Accepted

Chart

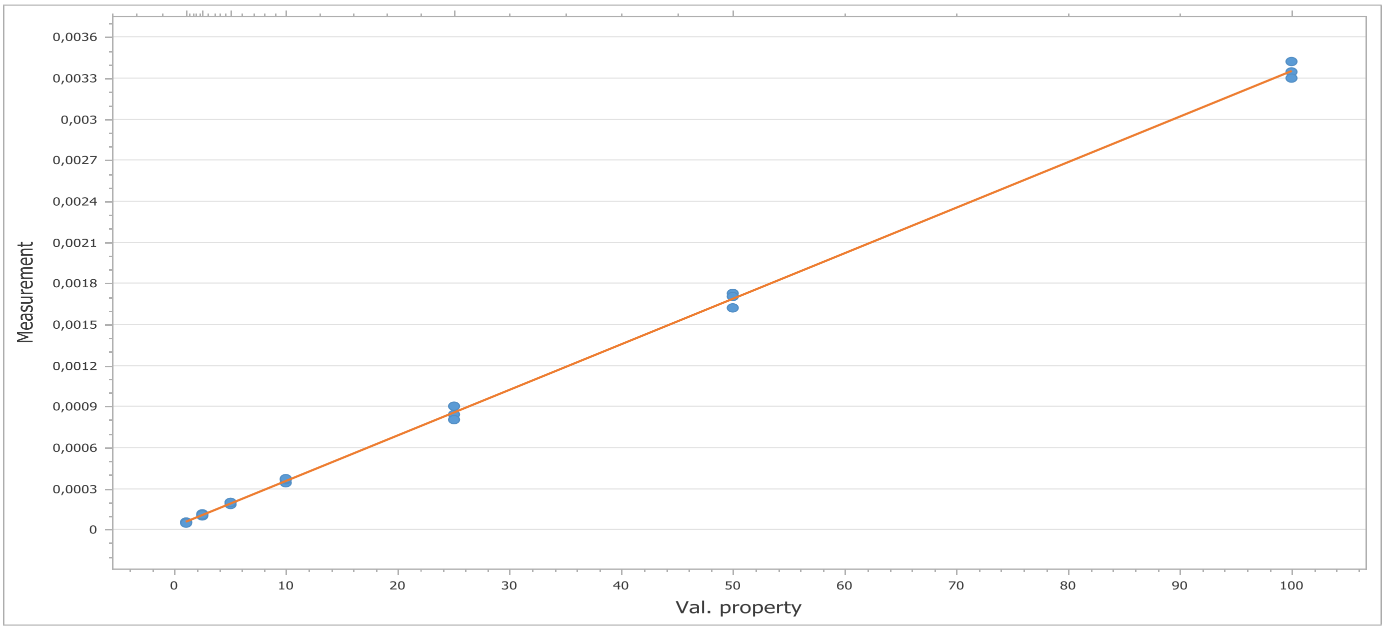

Conclusion: Linearity of the method was confirmed based on correlation and QC Coefficient.

The intercept value of the given straight line meets the acceptance criterion.

Straight line equation: y=2,3E-05+3,3E-05*x.

Supporting statistics: Intercept (estimate)= 2,3E-05

Slope (estimate)= 3,3E-05

Intercept standard deviation (estimate)= 9E-06

Slope standard deviation (estimate)= 0

Confidence interval for intercept= (4E-06) - (4,2E-05)

Confidence interval for slope= (3,3E-05) - (3,4E-05)

Residual variance (estimate)= 0

Residual standard deviation (estimate)= 3,1E-05

Correlation coefficient= 0,999651

R^2= 0,999301

QC coefficient= 3,213863

Entered data

Description – Calibration Solution	Val. property – Concentration [µg/L]	Measurement – Peak Area Ratio [1]
Calibration_Standard_001_µg/L	1	0.000301220885984832
Calibration_Standard_001_µg/L	1	0.000315590668612175
Calibration_Standard_001_µg/L	1	0.000275743489368293
Calibration_Standard_002.5_µg/L	2.5	0.00092530804784865
Calibration_Standard_002.5_µg/L	2.5	0.000894601971021729
Calibration_Standard_002.5_µg/L	2.5	0.000913067817410539
Calibration_Standard_005_µg/L	5	0.00196188948727619
Calibration_Standard_005_µg/L	5	0.00190437387626083
Calibration_Standard_005_µg/L	5	0.00191462602449669
Calibration_Standard_010_µg/L	10	0.00351571137708713
Calibration_Standard_010_µg/L	10	0.0038274881571974
Calibration_Standard_010_µg/L	10	0.00364961377472648
Calibration_Standard_025_µg/L	25	0.00880773492972636
Calibration_Standard_025_µg/L	25	0.0086131372408475
Calibration_Standard_025_µg/L	25	0.00880589278576951
Calibration_Standard_050_µg/L	50	0.0172301166295097
Calibration_Standard_050_µg/L	50	0.0180830175733046
Calibration_Standard_050_µg/L	50	0.0173552899063361
Calibration_Standard_100_µg/L	100	0.0377538175596685
Calibration_Standard_100_µg/L	100	0.0357558914264989
Calibration_Standard_100_µg/L	100	0.0374900518453799
Calibration_Standard_250_µg/L	250	0.0891459727316384
Calibration_Standard_250_µg/L	250	0.0918171696884097
Calibration_Standard_250_µg/L	250	0.092211412997743
Calibration_Standard_500_µg/L	500	0.184737935922539
Calibration_Standard_500_µg/L	500	0.182856711040399
Calibration_Standard_500_µg/L	500	0.18093938107748

Results

Calculated R	R^2	Reference R	Calculated QC	Reference QC	Max. acceptable intercept (absolute)	Calculated intercept value	Hypothesis
0.9999	0.9998	0.99	2.198	5	20	-0.0001	Accepted

Chart

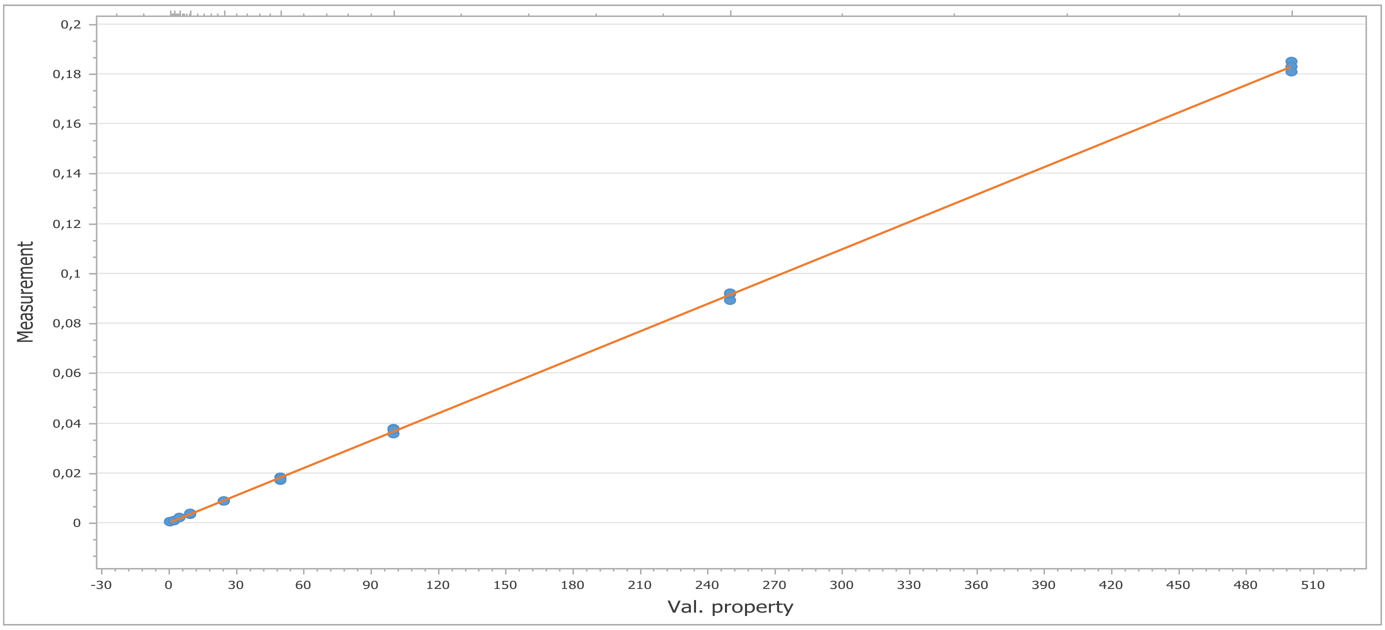

Conclusion: Linearity of the method was confirmed based on correlation and QC Coefficient.

The intercept value of the given straight line meets the acceptance criterion.

Straight line equation: y=-0.0001+0.0004*x.

Supporting statistics: Intercept (estimate)= -0.00012

Slope (estimate)= 0.000366

Intercept standard deviation (estimate)= 0.000201

Slope standard deviation (estimate)= 1E-06

Confidence interval for intercept= (-0.000535) - (0.000294)

Confidence interval for slope= (0.000364) - (0.000368)

Residual variance (estimate)= 1E-06

Residual standard deviation (estimate)= 0.000857

Correlation coefficient= 0.999899

R^2= 0.999798

QC coefficient= 2.197955
