# Supplementary material for: Development and validation of an LC-MS/MS method for determination of B vitamins and some its derivatives in whole blood
Source: PLoS One. 2022 Jul 14;17(7):e0271444. doi: 10.1371/journal.pone.0271444 (PMC9282541; doi:10.1371/journal.pone.0271444)
Supplement: Attachment — Submitted filename: Response to Reviewers.docx [file pone.0271444.s002.docx]

**Response to reviewers' comments - manuscript: PONE-D-22-10162**

**Reviewer #1:** The manuscript by David Kahoun et al. is an interesting LC-MS/MS study for quantification of various vitamins B in whole blood. The work is well-structured, well-written and easy to understand. However, the references are not sufficiently updated.:

1. Authors could improve the introduction section citing some recent studies for the analysis of various matrices: (these are only some receent examples)

• Roelofsen-de Beer RJAC, van Zelst BD, Wardle R, Kooij PG, de Rijke YB. Simultaneous measurement of whole blood vitamin B1 and vitamin B6 using LC-ESI-MS/MS. J Chromatogr B Analyt Technol Biomed Life Sci. 2017 Sep 15;1063:67-73. doi: 10.1016/j.jchromb.2017.08.011. Epub 2017 Aug 12. PMID: 28846867.

• Nguyen QH, Hoang AQ, Truong TMH, Dinh TD, Le TT, Luu THT, Dinh VC, Nguyen TMT, Vu TT, Nguyen TAH. Development of Simple Analytical Method for B-Group Vitamins in Nutritional Products: Enzymatic Digestion and UPLC-MS/MS Quantification. J Anal Methods Chem. 2021 May 5;2021:5526882. doi: 10.1155/2021/5526882. PMID: 34035973; PMCID: PMC8116160.

Authors: The introduction section has been updated with recent studies. Thank you for the notice.

**Reviewer #2:** In the manuscript titled “Development and validation of an LC-MS/MS method for determination of B vitamins and some its derivatives in whole blood”, Kahoun et al. present a LC-MS/MS based mass spectrometry method to examine multiple B-vitamins (and derivatives) within vertebrate samples. Specific comments are as follows:

1. Please provide a data supplement to show the calibration curves for each examined B vitamin (and derivative).

Authors: The required data supplement was added (S1 Calibration Curves). Thank you for the important suggestion.

2. While it appears that the zinc sulfate-based precipitation was more advantageous, a figure showing the direct comparison of the method vs. TCA is missing. Please provide plots showing the correlation of each B vitamin (and derivatives) between both methods.

Authors: The required plots were added as Fig 2 and Fig 3. Thank you for improving the visual presentation of our results.

3. For Table 5, provide information for age, sex, and n-value.

Authors: The required information is provided in the Table 5. Thank you for your request to fill in these important missing data.

4. Some modest changes to language are needed.

Authors: The required language changes were made. Thank you for the thorough language check of the text.
